# Contribution of COVID-19 to the Total Cases of Pulmonary Embolism and the Potential Risk Factors: Single Academic Hospital Study

**DOI:** 10.7759/cureus.29513

**Published:** 2022-09-23

**Authors:** Fawaz Altuwaijri, Karam Amshan, Amani Y Alanazi, Dalal F Alanazi, Hesham Alghofili, Mansour Altuwaijri, Talal Altuwaijri, Abdulmajeed Altoijry

**Affiliations:** 1 Department of Emergency Medicine, College of Medicine, King Saud University, Riyadh, SAU; 2 Division of Vascular Surgery, Department of Surgery, College of Medicine, King Saud University, Riyadh, SAU; 3 College of Medicine, Vision Colleges, Riyadh, SAU; 4 Division of Gastroenterology, Department of Medicine, College of Medicine, King Khalid University Hospital, King Saud University, Riyadh, SAU

**Keywords:** corona virus disease, sars-cov-2, pulmonary embolism < respiratory medicine, acute pulmonary embolism, covid-19

## Abstract

Background: There are limited data regarding potential triggering factors of pulmonary embolism (PE) in coronavirus disease 2019 (COVID-19) patients and its outcomes in comparison with non-infected PE patients. We aimed to identify the contribution of COVID-19 among patients diagnosed with PE and compare risk factors, laboratory results, and outcomes between COVID-19 PE patients and non-COVID-19 PE patients.

Methods: This was a retrospective study of all PE patients between March 2020 and December 2020. The patients were segmented into two groups based on a COVID-19 nasopharyngeal swab result. Statistical analysis was used to determine the differences in risk factors, laboratory values, and outcomes.

Results: A total of 58 patients were included. Females comprised 44.8% of the total sample. Overall, 16 patients (27.6%) were COVID-19 positive. Being non-Saudi was observed more in PE COVID-19 patients compared with non-COVID-19 patients (43.7% vs 4.8%, P = 0.001). Intensive care unit (ICU) admission occurred in 50% of COVID-19 PE patients.

Conclusion: COVID-19 was associated with 27.6% of the PEs in our hospital. Being male or a foreign resident was observed to be associated with COVID-19 PE. Further studies with larger sample sizes are needed, but these results may help the medical community regarding the increased risk of PE among COVID-19 patients and provide evidence of some potentially predictive factors that can be used to identify COVID-19 in high-risk patients.

## Introduction

A pulmonary embolism (PE) is diagnosed when a blood clot lodges in an artery supplying the lungs, resulting in an interruption of the blood flow to a lung lobe, segment, or part of a segment. PE is becoming a recognized complication of the coronavirus disease 2019 (COVID-19) [1]. There are an increasing number of studies reporting abnormal serum coagulation parameters in COVID-19 patients, mainly in hospitalized and intensive care unit (ICU) patients [1]. The receptor for SARS-CoV-2 (angiotensin-converting enzyme 2) is expressed on the membrane of the vascular muscle and endothelial cells, facilitating the formation of local thrombi [2]. Computed tomography (CT) has played a major role in the diagnosis of COVID-19 since the initial discovery of the disease and has helped to identify those patients with severe complications, including PE [3,4]. A recent retrospective cohort study reported an incidence rate of venothromboembolism (VTE) of 25%, with 10% of patients with COVID-19 dying of a VTE-related event [5]. The clinical relationship between PE and COVID-19 is already known [1]. However, data identifying risk factors for the prediction of PE in COVID-19 are limited. This study aimed to identify the contribution of COVID-19 to the total cases diagnosed with PE and compare risk factors, laboratory results, and outcomes in COVID-19 PE patients and non-COVID-19 PE patients.

## Materials and methods

Ethical consideration

This study was performed after obtaining approval from the King Saud University Institutional Review Board and was conducted in accordance with international research ethics standards. Patients provided informed consent. Project number: 21/01075/IRB.

Study subjects and settings

We reviewed all CT pulmonary angiogram (CTPA) scan examinations performed at King Saud University Medical City from March 1, 2020, to December 31, 2020. Images were obtained from the picture archiving and communication system database. We included all patients diagnosed with PE and segmented them into a COVID-19 positive group and a COVID-19 negative group based on reverse transcriptase polymerase chain reaction (PCR) results from nasopharyngeal swabs. To eliminate bias in the COVID-19 positive group, the group included two types of patients: (1) patients who came to the hospital with COVID-19 and then developed PE in the hospital; or (2) patients who came with PE and their admission swab turned out to be positive. Patients without a PCR test at admission were excluded (n = 2). All CTPA studies were initially read by radiology consultants, all with experience and not the authors of this study. The radiology reports for these examinations were reviewed to determine the presence or absence of PE. CT studies that were limited by respiratory motion or poor contrast opacification were excluded (n = 3). All patients had received VTE prophylaxis at admission (enoxaparin 40 mg daily, heparin 5000 U every eight hours, or sequential compression devices if anticoagulant prophylaxis was contraindicated). We compared risk factors, laboratory values, and PE outcomes for both groups. Variables included age, sex, nationality, and body mass index (BMI). Presence or history of smoking, deep venous thrombosis (DVT), previous PE, cancer, diabetes, heart failure, surgery within four weeks, hypertension, and a history of oral contraceptive use. Laboratory results included D-dimer levels, C-reactive protein, lactate dehydrogenase, and ferritin levels. Duration of ICU and ward admissions were also included. The outcome was assessed by the need for intubation and death announcement.

Statistical analysis

The Statistical Package for the Social Sciences for macOS (version 28.0; IBM Corp., Armonk, NY, USA) was used to analyze the results. Continuous variables are presented as the mean with a standard deviation. Categorical variables are presented as percentages. The χ² test of independence was used to determine the significance of the relationship between categorical variables. A Fisher exact test was used when variable frequencies were less than 5. A T-test was used to compare the means of continuous variables. P-values of less than 0.05 were considered significant. The odds ratio (OR) with a 95% confidence interval (CI) was calculated by multivariate analysis using logistic regression was used for COVID-19 positive group predictors.

## Results

A total of 58 patients were diagnosed with PE during the study period. All patients were tested for COVID-19. Females comprised (44.8%, n = 26) of the total sample. The average ages of both groups were comparable (Table 1). Patients with a BMI greater than 30 kg/m^2^ were seen more frequently in the COVID-19 negative group than in the COVID-19 positive group (63.16% vs 36.84%, n = 15 vs 7, respectively).

**Table 1:** Patient demographics and potential risk factors SD: standard deviation; OR; odds ratio; CI: confidence interval; n: number of patients; BMI: body mass index; DM: diabetes mellitus; HTN: hypertension; DVT: deep venous thrombosis; PE: pulmonary embolism; OCP: oral contraceptive pill.

Variable	COVID-19 positive n = 16 (27.6%)	COVID-19 negative n = 42 (72.4%)	P-Value
Age, y, mean ± SD	57.31 ±14.50	53.09 ±23.88	0.505
Gender, n (%)			
Male	14 (87.5)	18 (42.9)	0.006
Female	2 (12.5)	24 (57.1)	
BMI >30 kg/m^2^, n (%)	7 (43.8)	15 (35.7)	0.8
Nationality, n (%)			
Non-Saudi	7 (43.8)	2 (4.8)	0.001
Saudi	9 (56.3)	40 (95.2)	
History of, n (%)			
Smoking	3 (18.8)	2 (4.8)	0.114
DM	6 (37.5)	14 (33.3)	0.766
HTN	6 (37.5)	15 (35.7)	0.899
DVT	3 (18.8)	7 (16.7)	0.851
Previous PE	0 (0)	3 (7.1)	
Cancer	1 (6.3)	16 (38.1)	0.017
Heart failure	1 (6.3)	2 (4.8)	0.820
Surgery within four weeks	0 (0)	7 (16.7)	
OCP use	0 (0)	1 (2.4)	

Prevalence and potential risk factors

Among the 58 PE patients, 16 (27.6%) were found to be COVID-19 positive. Males were more likely to be seen in the COVID-19-positive group than the other groups (87.5% vs 42.9%, n = 14 vs 18, P = 0.006). Non-Saudis were more in the COVID-19 positive group than in the COVID-19 negative group (43.8% vs 4.8%, n = 7 vs 2, P = 0.001). Cancer was less frequently seen in the COVID-19 positive group than in the non-COVID-19 group (6.3% vs 38.1%, n = 1 vs 16, P = 0.017). There was no statistically significant difference in age, smoking, history of heart failure, history of PE, and diabetes or hypertension. Logistic regression analysis revealed a significant association between the presence of COVID-19 positive cases in PE patients and male gender [Beta = 2.41, OR = 11.14 (95% CI 1.15-107.87), P = 0.04], and being non-Saudi [Beta = 2.45, OR = 11.55 (95% CI 1.13-117.7), P = 0.04]. When we further analyzed as to which nationality had the highest incidence of PE, we did not find any significant results (Tables 1-2).

**Table 2:** Logistic regression model for predictors of COVID-19 positive cases in PE patients SD: standard deviation; OR: odds ratio; CI: confidence interval; n: number of patients; BMI: body mass index; DM: diabetes mellitus; HTN: hypertension; DVT: deep venous thrombosis; PE: pulmonary embolism; OCP: oral contraceptive pill.

Variable	Beta	SD	Z-value	P-value	OR (95% CI)
Age	−0	0.03	0.02	0.98	1 (0.94–1.06)
Male gender	2.41	1.16	2.08	0.04	11.14 (1.15–107.87)
Non-Saudi	2.45	1.18	2.07	0.04	11.55 (1.13–117.7)
BMI >30 kg/m^2^	−0	0	0.06	0.95	1 (1–1)
History of					
Smoking	1.55	1.52	1.02	0.31	4.69 (0.24–91.39)
DM	1.53	1.41	1.09	0.28	4.62 (0.29–72.64)
HTN	-0.57	1.22	0.47	0.64	0.56 (0.05–6.12)
DVT	1.09	1.24	0.88	0.38	2.98 (0.26–33.67)
Previous PE	−1.89	2.44	0.77	0.44	0.15 (0–17.98)
Cancer	−2.81	1.68	1.67	0.09	0.06 (0–1.62)
Heart failure	−0.14	1.74	0.08	0.93	0.87 (0.03–26.41)
Surgery within 4 weeks	−2.32	1.98	1.17	0.24	0.1 (0–4.74)
OCP use	-0.15	13.76	0.01	0.99	0.86 (0–446.54)

Outcomes and laboratory values

From the time of admission, PE in COVID-19 patients was diagnosed earlier (1.29 ± 0.02 days) than in the COVID-19 negative group. In our population, we found that COVID-19-negative patients are likely to stay (7.58 ± 18.88 days) longer in the hospital when affected by PE in comparison with COVID-19 patients. COVID-19 patients tend to have more chances of ICU admission than COVID-19 negative patients (50% vs 33.3%, n = 8 vs 14, P = 0.246). There was no significant difference in laboratory values between the groups. The death ratio was higher in the PE COVID-19 group compared with the PE non-COVID (Table 3).

**Table 3:** Hospital stay, laboratory values, and outcomes SD: standard deviation; OR: odds ratio; CI: confidence interval; NA: -; n: number of patients; ICU: intensive care unit; CRP: C-reactive protein; LDH: lactate dehydrogenase.

Variable	COVID-19 positive n = 16 (27.6%)	COVID-19 negative n = 42 (72.4%)	P-value
Hospital stay until PE, days, mean ± SD	4.91 ± 5.17	6.20 ± 5.19	0.500
Total hospital stay, days, mean ± SD	11.83 ± 7.74	19.41 ± 26.62	0.375
ICU admission, n (%)	8 (50)	14 (33.3)	0.246
D-dimer level, μg/mL, mean ± SD	6.16 ± 6.22	22.94 ± 65.26	0.697
CRP, μg/mL, mean ± SD	76.54 ± 66.31	99.77 ± 108.40	0.235
LDH, IU/L, mean ± SD	529.4 ± 392.82	525 ± 379.91	0.465
Ferritin, ng/mL, mean ± SD	980.46 ± 1361.83	973.96 ± 1772.97	0.761
Intubation, n (%)	4 (25)	3 (7.14)	0.078
Death, n (%)	4 (25)	9 (21.4)	0.771

## Discussion

The incidence of PE in COVID-19 patients who undergo CTPA is reported to be between 20% and 30% [6,7]. Approximately 20% of COVID-19 patients develop PE [8]. In our study, we found that out of all PEs diagnosed, 27.6% were associated with COVID-19 infection. Our patients presented with symptoms like fever, cough, and shortness of breath, which are symptoms of PE caused by COVID-19 or by other causes [9,10]. In a study performed in our institution in 2011, females were at more risk of PE than their male counterparts with a ratio of 2:1, and that comes in line with the international trends [11,12]. However, when it comes to VTE events in COVID-19 patients, published reports show that the male gender predominates [13,14]. Our results match with prior publications [13,14]. Our results have shown that most of the non-Saudis seen in the PE COVID-19 positive group, from our observation, non-Saudi patients usually present late to the hospital when they are very sick, and this may be the explanation for this finding in our study. According to the Saudi General Authority of Statistics 2016 demographic survey, the socioeconomic status of non-Saudis is lower than Saudis and most of them are less educated [15]. Lower socioeconomic status is known to be associated with higher levels of venous thromboembolism events and worse outcomes, at both population and individual levels [16,17]. Moreover, there is a known disparity in healthcare delivery and hospital outcomes between Saudis and non-Saudis [18]. Prior to the pandemic, non-Saudis were required to pay out of pocket or have health insurance. However, in April 2020, the Saudi health authorities announced that COVID-19 treatment would be free for all citizens and residents. This includes residency violators without any legal consequences [19]. Hypercoagulability, increased blood viscosity, and inflammation are all VTE-provoking mechanisms of smoking [20]. Active smoking was found to be a predictor of mortality in critically ill COVID-19 patients [21]. Badr et al. study identified smoking as a risk factor for PE in COVID-19 patients [22]. The lack of a significant difference between the two groups in our study is mainly because smoking is a known risk factor for PE regardless of COVID-19 status. The low sample size might also play a significant role in that result. Obesity (BMI>30 kg/m^2^) in COVID-19 patients is known to cause severe manifestations of the disease [23]. Furthermore, obesity is associated with a 2.7-times increased risk of PE when compared with no PE in COVID-19 patients, which explains why we found that 43.8% of COVID-19 group patients were obese [24]. We did not find a significant difference in the length of hospital stay, ICU admission, or the need for intubation between the two groups. Nevertheless, COVID-19-negative patients tended to stay longer in the hospital, and this may be due to the fact that they had more severe comorbidities such as cancer, heart failure, and a history of PE. Our data suggest that COVID-19 patients can develop PE regardless of requiring ICU care (50% did not require ICU care), which is in contrast to Griller et al. and in line with Poyiadji et al. [6,24]. The noticeable changes in blood coagulation during COVID-19 infection have been well documented and include increased values of D-dimer, fibrin or fibrinogen degradation products, and increased fibrinogen and decreased antithrombin values, prothrombin time activity, and thrombin time [25]. A systemic pro-inflammatory cytokine response is the main mediator of thromboembolism events, inducing the expression of procoagulant factors, local inflammation, and hemodynamic alterations [2]. Clinically, both groups had underlying inflammation. As a result, we found no statistically significant difference in laboratory results. The higher mortality rate in our cohort can be explained by the fact that 9 out of the 13 deaths occurred in cancer and heart failure patients. Cancer and heart failure are predictors of mortality in PE patients [26]. Our study results are limited by the retrospective study design and the constraint of a single-health center. Furthermore, the generalizability to other countries and races may not be applicable due to socioeconomic differences.

## Conclusions

In conclusion, COVID-19 is diagnosed in 27.6% of PE cases confirmed with CTPA, and this percentage has a dramatic impact on any healthcare system. Being male or non-Saudi was observed to be more common with COVID-19 PE. Non-Saudis were found more in the COVID-19 positive group versus the COVID-19 negative group. The longer length of stay was noted in our group of patients with PE who were COVID-19 negative, while COVID-19 patients required ICU care and mechanical ventilation. We found a mortality rate of one in every four COVID-19 patients in the study. No statistically significant difference was observed in laboratory values or other clinical outcomes. Further studies with larger sample sizes are encouraged to enhance the statistical power.
